# The alcohol industry, charities and policy influence in the UK

**DOI:** 10.1093/eurpub/cku076

**Published:** 2014-06-09

**Authors:** Sarah M Lyness, Jim McCambridge

**Affiliations:** London School of Hygiene and Tropical Medicine, London, UK

## Abstract

**Background:** Charities exist to pursue a public benefit, whereas corporations serve the interests of their shareholders. The alcohol industry uses corporate social responsibility activities to further its interests in influencing alcohol policy. Many charities also seek to influence alcohol and other policy. The aim of this study was to explore relationships between the alcohol industry and charities in the UK and whether these relationships may be used as a method of influencing alcohol policy. **Methods:** The charity regulator websites for England and Wales and for Scotland were the main data sources used to identify charities involved in UK alcohol policy making processes and/or funded by the alcohol industry. **Results:** Five charities were identified that both receive alcohol industry funding and are active in UK alcohol policy processes: Drinkaware; the Robertson Trust; British Institute of Innkeeping; Mentor UK and Addaction. The latter two are the sole remaining non-industry non-governmental members of the controversial responsibility deal alcohol network, from which all other public health interests have resigned. **Conclusion:** This study raises questions about the extent to which the alcohol industry is using UK charities as vehicles to further their own interests in UK alcohol policy. Mechanisms of industry influence in alcohol policy making globally is an important target for further investigations designed to assist the implementation of evidenced-based policies.

## Introduction

Alcoholic beverage producers, like all corporations, exist ultimately to serve the interests of their shareholders. A key component of corporate strategy is to avoid an unfavourable regulatory environment by influencing policy.^1^^,^^2^ The concentration of the alcohol industry globally into a small number of large and powerful alcohol corporations has increased the capacity to influence national governments.^3^

The alcohol industry’s interests are not monolithic^4^ and are represented by a range of organizations including traditional trade bodies and, more recently, by industry-funded ‘social aspects and public relations organisations’ (SAPROs).^5–7^ SAPROs manage issues that may be detrimental to the alcohol industry.^6^ The Portman Group, for example, was established by the industry in 1989 and is well placed in UK policy circles.^8^ It set up the Drinkaware website, and with the agreement of a range of governmental agencies, constituted this as a charity, The Drinkaware Trust, in 2006^9^ (Supplementary ref. w1). Justifications such as ‘corporate social responsibility’ and ‘partnerships with the public health community’ are used for furthering the industry’s economic interests in policy making.^5^ Little attention has been previously given to industry funding and engagement with charities, with the exception of Drinkaware.^9^

The 2004 alcohol policy for England^10^ was strongly criticized for not being evidence based, likely to be ineffective and dominated by industry interests.^11–13^ Subsequent policy statements by the 1997–2010 Labour Government were criticized on similar grounds.^14^ After deciding in 2012 to adopt a key evidence-based policy measure, minimum unit pricing (MUP), the current conservative-led UK government halted its implementation in 2013,^15^^,^^16^ amidst widespread speculation about alcohol industry influence (Supplementary ref. w2).

In 2010, the newly elected UK government’s approach to public health policy making was to work collaboratively with the alcohol and other industries, as well as with health charities and other organizations, within the Public Health Responsibility Deal (PHRD; Supplementary ref. w3). Policy areas within the PHRD include alcohol, diet and physical activity. Although excluding the tobacco industry, the involvement of alcohol and other industries has been heavily criticized^17^ and has led to a series of withdrawals of public health interests, most recently over MUP (Supplementary refs w4–w6).

Charities exist to pursue a public benefit and must have a charitable purpose as defined in legislation, such as the advancement of health.^18^ They are controlled by trustees (Supplementary ref. w7) who must follow specific governance and reporting requirements. Charities with turnover over £5000 must be registered with and regulated by the Charity Commission.^18^ The website of the Charity Commission for England & Wales contains details of all registered charities, including the past 4-year annual report and accounts for each registered charity with income over £25 000 (Supplementary ref. w8). Similar arrangements exist within Scotland, although the Scottish website includes less information about each charity. A register of charities is currently being set up in Northern Ireland (Supplementary ref. w9).

Not for profit organizations can easily be confused with charities. Although charities are the most common type of not for profit organizations, many are not charities and so are not regulated by the Charity Commission (Supplementary ref. w10). The Portman Group (Supplementary ref. w11) and think tanks such as the Adam Smith Institute (Supplementary ref. w12) are examples of not for profit organizations that are not charities.

This was an exploratory study to establish what can be learnt about the alcohol industry’s relationships with charities in light of apparent industry influence on policy in England.^19^ We particularly sought to identify evidence of funding or other associations with charities that may suggest attempts to influence UK alcohol policy. We used the publicly available charity regulator websites as the primary data sources.

## Methods

Data collection proceeded iteratively and was undertaken by the first author unless otherwise indicated. The Charity Commission website for England and Wales (Supplementary ref. w8) was searched in November 2013. Search terms used were ‘alcohol’, ‘addiction’, ‘drug’, ‘drugs’ and ‘dependence’ in name, charitable objects and activities fields. This was designed to return results for charities where alcohol was a significant part of their work, according to charities own descriptions. Name, charitable objects and activities are all set by the charities themselves. Charitable objects state the purpose of the charity and form part of the registration process. Describing charitable activities are part of the annual reporting requirements. All charities with annual incomes over £10M in the most recent annual report and accounts (includes trustees report and audited accounts) were examined to identify income from the alcohol industry and UK alcohol policy-related activities (see table 1 for examples). Charities with annual incomes £0.5M–£10M were examined by the second author for involvement in alcohol policy or industry links. Funding thresholds were designed to identify charities with potential to be actively trying to influence UK alcohol policy.

**Table 1:** Charities receiving alcohol industry funding and involved in UK alcohol policy

Charity	Income^a^	Alcohol industry funding	Examples of alcohol policy involvement
Drinkaware	£5.1M (Supplementary ref. w13)	98% funding from alcohol industry, the remainder including the sale of publications to the public sector (Supplementary ref. w13)	Presented evidence to health select committee on governments alcohol strategy (Supplementary ref. w13)
The Robertson Trust	£20.6M (Supplementary ref. w22) Funded Mentor UK (Supplementary ref. w14)	Owns Edrington, maker of major whisky brands (including Famous Grouse, Cutty Sark). Trust income derives from Edrington’s profits (Supplementary ref. w14).	None direct found for Robertson Trust. Edrington responded to government consultations^21^ (Supplementary ref. w23)
British Institute of Innkeeping	£4.2M (Supplementary ref. w15)	Income from individual and corporate membership and qualification/training fees (Supplementary ref. w15)	Responded to Home Office consultations (Supplementary refs w24,w25)
Addaction	£46.9M (Supplementary ref. w18)	Two projects funded by Heineken UK—£351k in 2011/12 (Supplementary ref. w18) and £80k in 2010/11 (Supplementary ref. w26) and undisclosed amount in 2009/10 (Supplementary ref. w27). £1M pledged by Asda (Supplementary ref. w18).	Core group member responsibility deal alcohol network (Supplementary ref. w28)
Mentor UK	£0.4M down from £1.3M in 2011/12 (Supplementary ref w19). Previous years £563k in 2010/11, £688k in 2009/10, £644k in 2008/09 (Supplementary refs w29–w31)	CHAMPS awards (see text) funded by Diageo £30k in 2011/12 (Supplementary ref. w19) £105k in 2010/11 (Supplementary ref. w29), £74k in 2009/10 (Supplementary ref. w30), £162k in 2008/09 (Supplementary ref. w31). Funding from Robertson Trust £50k in 2012/13 (Supplementary ref. w14) and Gannochy Trust in 2011/12 (Supplementary ref. w19)	Core group member responsibility deal alcohol network (Supplementary ref. w28)

^a^Most recent year available.

The charities identified through this process were further investigated by reviewing their last 4-year annual reports and accounts that were accessible. All trusts (typically charities who give grants to other charities) named as donors to these charities were investigated for alcohol links in two ways. First, the trust’s charitable objects and activities were identified on both the England and Wales and the Scottish charity regulator websites. Secondly, the trust’s own website was searched. Where an alcohol connection was found, the trust was added to the selected charities for further investigation as described above. During this process, other charities identified as receiving funding were selected for further data collection from alcohol industry and other websites.

The approach was repeated for Scotland, with the following variations due to the more limited search functionality, and the lack of annual report and accounts registered on the Scottish site. The Office of the Scottish Charity Regulator website (Supplementary ref. w9) was searched for name only. Charities were identified with income over £1M. The annual report and accounts were retrieved from the charities own website where available and analysed. Where the annual report and accounts were not available, the content of the website was searched. All search results below £1M income on the regulator website were examined by the second author as above. Funding thresholds were lower as smaller scale may be sufficient to seek policy influence in a less populous country with a more open style of policy making.^20^

## Results

The searches returned 1434 charities in England and Wales, of which 23 had incomes over £10M, and 197 incomes £0.5M–£10M, and 49 charities in Scotland of which 5 had income over £1M. The vast majority of these charities receive local government grants for service delivery and are not involved in alcohol policy.

### Industry-funded charities active in UK policy

Five charities were identified as active in UK alcohol policy and being alcohol industry funded (table 1). Three charities, Drinkaware, The Robertson Trust and British Institute of Innkeeping, receive almost all their income from the alcohol industry or from people working in the industry (Supplementary refs w13–w15) and have senior alcohol industry figures as trustees (Supplementary ref. w13, w16, w17). Two other charities, Addaction and Mentor UK receive industry funding alongside public sector grants, and are active in alcohol policy (Supplementary ref. w18, w19). Neither have alcohol industry figures as trustees (Supplementary refs w20, w21).

Drinkaware is funded by donations from the alcohol industry (Supplementary ref. w13). Previous investigations have identified problematic aspects of the information it provides to the public and to policy makers.^9^ Drinkaware is the only alcohol SAPRO that is a charity in the UK.

The Robertson Trust is unusual in controlling The Edrington Group, a major producer of whisky, and its income derives from the Edrington business (Supplementary refs w32, w33). The trust was originally formed by the Robertson family who were involved in distilling. It is a grant awarding trust, providing funding for a wide range of charities, including Mentor UK. The Robertson Trust and The Edrington Group shared a Chairman, Sir Ian Good, for more than a decade (Supplementary ref. w16). Although no evidence was found of the Robertson Trust itself being directly active in UK alcohol policy, The Edrington Group is active in the alcohol policy environment, for example making submissions to public consultations on MUP, the content of which are very similar to other industry submissions.^21^^,^^22^

The British Institute of Innkeeping is a membership organization for the licensed retail trade, offering qualifications, education and training to members, licensees and their staff (Supplementary ref. w15). Corporate members include spirits producers, such as Diageo and Bacardi Brown-Forum Brands, brewers such as Fullers and licensed retailers such as Wetherspoons (Supplementary refs w34, w35).

Addaction offers services to people affected by drug and alcohol problems (Supplementary ref. w18). It received 0.7% of its income in 2011/12 from Heineken to deliver ‘Mutual Aid Partnership’, a peer support group programme to help recovery from alcohol problems, and a ‘Resettlement Project’ for people leaving prison (Supplementary ref. w18). Other activities include ‘a commission investigating the resettlement of prisoners back into the community with an alcohol addiction’ (Supplementary ref. w36). Joint work between Addaction and Heineken has received wide publicity, and is promoted as evidence of successful partnership working (Supplementary refs w37, w38).

Mentor UK runs programmes to protect children from alcohol and drugs through mentoring and education (Supplementary ref. w19). In 2008/09 Mentor UK received 25% of its income from Diageo, and continues to receive funding for CHAMPS (Children’s Health through Alcohol Misuse Prevention), Mentor UK’s awards for projects with children (Supplementary ref. w19). Mentor UK received grants from both The Robertson Trust and The Gannochy Trust (see below) (Supplementary ref. w14, w19). Mentor UK is part of Mentor International that claims to be ‘the leading international non-government organisation working globally to prevent substance abuse’ (Supplementary ref. w39). Mentor International also receives funding from the Robertson Trust (Supplementary ref. w39).

Addaction and Mentor UK are now the sole charity representatives of the PHRD alcohol network (Supplementary ref. w28). All other charities and health bodies have withdrawn due to concerns about alcohol industry influence (Supplementary refs w4–w6). Both supported the introduction of MUP and were critical of the decision not to implement it, and justified their continued participation in the PHRD alcohol network (Supplementary refs w40, w41). Neither Addaction nor Mentor UK have secure long-term funding.

### Other charities industry funded or active in alcohol policy

In addition to the five charities that were both funded directly by the alcohol industry and active in UK policy, seven other charities were identified which were either alcohol industry funded or active in the policy environment, but not both.

Two charities, The Diageo Foundation and The Gannochy Trust, receive all their funding from the alcohol industry (Supplementary refs w42, w43), but are apparently inactive in alcohol policy. The Gannochy Trust is a Scottish grant giving trust, funded from the profits of Bells Whisky (Supplementary ref. w43). It gives grants to charities including Mentor UK (Supplementary ref. w19), but is not active in UK alcohol policy.

Diageo is one of the world’s largest producers of spirits. It is the only member of the Portman Group with its own charity. The charitable foundation is very closely aligned with Diageo, having trustees appointed by the Diageo Board (Supplementary ref. w44) and all of its income of £1.5M from Diageo in 2012 (Supplementary ref. w42). The Diageo Foundation gave 528 grants in 2012 to charities including Reading University for scholarships (£50k) and Cancer Research UK (£24k) (Supplementary ref. w42), but is not active in UK alcohol policy. Diageo Great Britain, the UK subsidiary of Diageo, directly funded Mentor UK until 2010 (Supplementary ref. w30) rather than funding through the charitable foundation. Diageo itself is very active in seeking to influence UK alcohol policy (Supplementary refs w45, w46).

Harm Reduction International [also known as International Harm Reduction Association (IHRA)] received funding from Diageo for an alcohol in the City Project for £42k in 2010 (Supplementary ref. w47). Despite declarations in the annual reports that it would not accept funding from the alcohol industry, £15k in donations was declared from ‘alcohol industries’ during 2008 (Supplementary ref. w48). IHRA’s principal focus has been on injecting drug use and HIV internationally, though they have included work on alcohol since 2004. Policy influence activities are at international rather than the UK level (Supplementary ref. w49) through various collaborations with the International Center for Alcohol Policies, the key international SAPRO.^23^ These have included conferences and publications promoting industry-favoured perspectives on alcohol policy (Supplementary ref. w50).

Four charities, Alcohol Concern, Alcohol Focus Scotland, the Society for the Study of Addiction (publisher of Addiction) and Alcohol Research UK are active in UK alcohol policy or debates (Supplementary refs w51–w54). Alcohol Concern, Alcohol Focus Scotland and Alcohol Research UK have stated policies of not accepting alcohol industry funds (Supplementary refs w55–w57), though the latter has received income from Drinkaware for one project (Supplementary ref. w54). Alcohol Concern and Alcohol Focus Scotland have recently experienced reductions in grant income from government funding (Supplementary refs w52, w58).

## Discussion

This study has revealed inter-relationships between the alcohol industry and charities active in UK alcohol policy. The only two remaining charities engaged in the PHRD alcohol network, Addaction and Mentor UK, have accepted funding from various alcohol industry sources. Three other charities are almost entirely funded by the alcohol industry. Drinkaware promotes information and education rather than evidence-based policies such as MUP,^9^ while British Institute of Innkeeping is an overt industry body, drawing its membership from the industry. The Robertson Trust owns a major spirits producer.

As the first study to use the charity regulators’ websites as the primary data source to investigate the alcohol industry, it is important to recognize the limitations of this study. Although charities are required to complete annual reports and accounts with specific reporting requirements, these do not include the mandatory disclosure of funding sources, thus permitting confidentiality for donors. The years for which we were able to capture data are necessarily limited and although we found some disclosures of alcohol industry funding, there may well be additional funding that is not declared, simply because it is not required to be so.

There are other charities active in the alcohol policy arena where alcohol is less prominent in their activities, such as charities focussing on specific diseases including heart disease and cancer and the Royal Colleges. These were excluded from this study, as were smaller charities in England and Wales. This is important to note as one recent study has identified think tanks, some of whom are charities, are vehicles of alcohol industry influence in the UK.^24^

The website of the Office of Scottish Charity Regulator has more limited functionality than that for England and Wales, without the ability to search charitable objects or activities, and without posting the registered charities’ annual report and accounts. Many charities do not publish them online and instead release them only when requested.

This study poses interesting questions about whether the alcohol industry is using charities to further their own interests. The headquarters of Addaction and Mentor UK are co-located above a pub. Both charities have accepted alcohol industry funding and have remained engaged in the controversial PHRD Alcohol Network. The types of treatment and prevention work undertaken by both organizations are unthreatening to the economic interests of the alcohol industry. It is possible that both these decisions reflect a core philosophy within the charities of engagement with industry, but it is also possible that the receipt of funding itself has influenced the decision to remain in the PHRD. Regardless of the validity of these candidate explanations, industry influence is a matter of obvious concern.

Three of the five identified charities have relatively secure income from industry: The Robertson Trust has an endowment; Drinkaware receives income direct from the alcohol industry; and the British Institute of Innkeeping’s income comes from both membership fees and commercial operations. For other charities, when income from public donations and public sector sources is squeezed, corporate funding may become more attractive, making them vulnerable to corporate influence. Corporations gain directly from public relations benefits, and indirectly if the policy influencing activities of charities are shaped by funding (Supplementary ref. w59). It is well established that the tobacco industry has used corporate philanthropy as a political device,^25–27^ though little attention internationally has previously been paid to the alcohol industry and charities.^28^^,^^29^ Attention is also shifting to other strategies used by the tobacco industry, such as subversion of peer-reviewed science.^30–32^

The Robertson Trust’s control of, makes it the owner of, The Edrington Group, and it should be considered to be part of the alcohol industry. The relationship between The Edrington Group and The Robertson Trust is, however, somewhat opaque; it is not obvious how far The Robertson Trust controls the Edrington Group or vice versa.

The charity regulator websites have proved to be a useful research resource here. This study reveals the complex nature of the links between the alcohol industry and charities, though unavoidably this picture is incomplete. Whether and to what extent industry support for charities is an attempt to buy influence either within the charity or within the alcohol policy-making environment needs further study. Charities operating in alcohol or other policy arenas should be required to declare any possible conflicts of interest from funding sources, to ensure greater transparency. Such a requirement should also apply to other actors, so that for example the economic motivations of corporate policy influencing activities should be taken into account, alongside claims of corporate social responsibility.

Charities core purpose is to work for and support public benefit. Corporations are legally required to serve the interests of their shareholders. A key component of corporate strategy is doing what is necessary, within the law, to influence policy, directly and indirectly and both openly and behind closed doors.^33^ Unlike work on tobacco control, alcohol researchers are largely restricted to using publicly available data sources to attempt to gain insights into the mechanics of policy influence, including the use of charities.^34^ Policy makers in the UK and elsewhere should consider whether current levels of transparency in policy making best serve public benefit.

## Supplementary Material

Supplementary Data
