# Supplementary material for: The alcohol industry, charities and policy influence in the UK
Source: Eur J Public Health. 2014 Jun 9;24(4):557–61. doi: 10.1093/eurpub/cku076 (PMC4110957; doi:10.1093/eurpub/cku076)
Supplement: Supplementary Data [file supp_24_4_557__index.html]

The alcohol industry, charities and policy influence in the UK — The alcohol industry, charities and policy influence in the UK — Supplementary Data 

# The alcohol industry, charities and policy influence in the UK

## Supplementary Data

files

**Files in this Data Supplement:**

- Supplementary Data - docx file
