# Supplementary material for: The alcohol industry, charities and policy influence in the UK
Source: Eur J Public Health. 2014 Jun 9;24(4):557–61. doi: 10.1093/eurpub/cku076 (PMC4110957; doi:10.1093/eurpub/cku076)
Supplement: Supplementary Data [file supp_cku076_ejph-2014-02-om-0102-File002.docx]

## Supplementary Material

## Web Based References

w1. Portman Group. Portman Group - History and Mission [Internet]. 2013 [cited 2013 Nov 25]. Available from: http://www.portmangroup.org.uk/about. Archived at: http://www.webcitation.org/6LOLUQxDD.

w2. Boseley S. Minimum unit price for alcohol proposal shelved | Society | The Guardian [Internet]. Guardian. 2013 [cited 2013 Dec 2]. Available from: http://www.theguardian.com/society/2013/jul/17/minimum-unit-price-alcohol-shelved. Archived at http://www.webcitation.org/6LZL05Aco.

w3. Department of Health. About the Public Health Responsibility Deal [Internet]. Department of Health; 2013 [cited 2013 Dec 2]. Available from: https://responsibilitydeal.dh.gov.uk/about/. Archived at: http://www.webcitation.org/6LYt8TUOq.

w4. Cancer Research UK, Faculty of Public Health, UK Health Forum, Sheron N. NGOs pull out of Government’s Alcohol Network of the Responsibility Deal [Internet]. Fac. Public Heal. website. 2013 [cited 2013 Nov 19]. Available from: http://www.fph.org.uk/ngos_pull_out_of_government’s_alcohol_network_of_the_responsibility_deal. Archived at: http://www.webcitation.org/6LFCyCBEX.

w5. Royal College of Physians, British Association for the Study of the Liver, British Liver Trust, Studies BMAI of A. Key health organisations do not sign responsibility deal [Internet]. RCP Website. London; 2011 [cited 2013 Nov 19]. Available from: http://www.rcplondon.ac.uk/press-releases/key-health-organisations-do-not-sign-responsibility-deal. Archived at: http://www.webcitation.org/6LFCQrDA2.

w6. Alcohol Concern. Letter from Alcohol Concern to Secretary of State for Health re Responsibility Deal [Internet]. London; 2011. Archived at: http://www.webcitation.org/6L4b4qjJC

w7. Charity Commission. Becoming a trustee - Charity Commission [Internet]. 2013 [cited 2013 Nov 18]. Available from: http://www.charitycommission.gov.uk/trustees-staff-and-volunteers/trustee-role/becoming-a-trustee/. Archived at: http://www.webcitation.org/6LE2PDvYN.

w8. Charity Commission. Home - Charity Commission [Internet]. 2013 [cited 2013 Nov 6]. Available from: http://www.charitycommission.gov.uk/. Archived at: http://www.webcitation.org/6KvnGngh6.

w9. Office of the Scottish Charity Regulator. OSCR - Office of the Scottish Charity Regulator [Internet]; 2013 [cited 2013 Nov 6]. Available from: http://www.oscr.org.uk/. Archived at: http://www.webcitation.org/6KvoQ4ZXO

w10. Jones E, Smith J. Not-For-Profit Operations [Internet]. London; 2012. Available from: http://www.webcitation.org/6LONIUYe7. Archived at http://www.webcitation.org/6LONIUYe7.

w11. Portman Group. House of Commons - Health Committee: Written evidence from The Portman Group (GAS 54) [Internet]. 2012. Archived at: http://www.webcitation.org/6LOOPEiLB.

w12. Adam Smith Institute. About us | Adam Smith Institute [Internet]. 2013 [cited 2013 Nov 25]. Available from: http://www.adamsmith.org/about-us. Archived at: http://www.webcitation.org/6LOP4QW0H.

w13. The Drinkaware Trust. Annual Report and Financial Statements 2012 [Internet]. London; 2012. Archived at: http://www.webcitation.org/6L3PKPMPr.

w14. The Robertson Trust. The Robertson Trust Annual Review 2013. 2013; Archived at: http://www.webcitation.org/6L6MsnGDz.

w15. British Institute of Innkeeping. The British Institute of Innkeeping Report & Financial Statements 2012 [Internet]. Camberley; 2012. Archived at: http://www.webcitation.org/6LPkbWNW1.

w16. McConnell I. Former Edrington chairman has had a Good run at the top | Herald Scotland [Internet]. Her. Scotl. 2013 [cited 2013 Nov 13]. Available from: http://www.heraldscotland.com/business/people/former-edrington-chairman-has-had-a-good-run-at-the-top.21926692. Archived at: http://www.webcitation.org/6L6OJQxme.

w17. British Institute of Innkeeping. BII’s charitable status - BII [Internet]. 2013 [cited 2013 Nov 25]. Available from: http://www.bii.org/about-bii/charitable-status. Archived at: http://www.webcitation.org/6LPnNz1RP.

w18. Addaction. Addaction Report and Financial Statement 2012 [Internet]. London; 2012. Archived at: http://www.webcitation.org/6L3IOpsZL.

w19. Mentor Foundation UK. Mentor UK Annual Report and Financial Statements 2012 [Internet]. 2012. Archived at: http://www.webcitation.org/6L3Ne6soR.

w20. Addaction. Addaction - Board of Trustees [Internet]. 2013 [cited 2013 Nov 11]. Available from: http://www.addaction.org.uk/personnel.asp?section=137&sectionTitle=Board+of+Trustees. Archived at: http://www.webcitation.org/6L3LaIgez.

w21. Mentor UK. Mentor UK Trustees [Internet]. 2013. Available from: http://www.mentoruk.org.uk/about-us/who-we-are/trustees/. Archived at: http://www.webcitation.org/6L3Og0PAz.

w22. Office of the Scottish Charity Regulator. Extract from the Scottish Charity Register - Robertson Trust; 2013 [cited 2013 Nov 13]; Available from: https://www.oscr.org.uk/search-charity-register/charity-extract/?charitynumber=sc002970. Archived at: http://www.webcitation.org/6L6M2nBSF.

w23. The Edrington Group. Scottish Government Strategy for Alcohol : The Edrington Group Response [Internet]. Archived at: http://www.webcitation.org/6LaWF5o2U

w24. British Institute of Innkeeping. Response to Rebalancing of the Licensing Act - BII [Internet]. 2010. Available from: https://www.bii.org/documents/647. Archived at: http://www.webcitation.org/6LPmMkQfY.

w25. British Institute of Innkeeping. Drink Driving Consultation Response - BII [Internet]. 2010. Available from: http://www.bii.org/documents/644. Archived at: http://www.webcitation.org/6LPmZ78mp.

w26. Addaction. Addaction Report and Financial Statements 2011 [Internet]. London; 2011. Archived at: http://www.webcitation.org/6L62CMenj

w27. Addaction. Addaction Report and Financial Statements 2010 [Internet]. London; 2010. Archived at: http://www.webcitation.org/6L63HTnpP

w28. Department of Health. Alcohol network’s core group [Internet]. Department of Health; 2013 [cited 2013 Nov 11]. Available from: https://responsibilitydeal.dh.gov.uk/alcohol-network-core-group/. Archived at: http://www.webcitation.org/6L3L5SiR8

w29. Mentor Foundation UK. Mentor Foundation UK Trustees Report 2011 [Internet]. London; 2011. Archived at: http://www.webcitation.org/6L65JeCpb

w30. Mentor Foundation UK. Mentor Foundation Trustees Report and Accounts 2010 [Internet]. London; 2010. Archived at: http://www.webcitation.org/6L6923JNw

w31. Mentor Foundation UK. Mentor Foundation UK Trustees Report and Accounts 2009 [Internet]. London; 2009. Archived at: http://www.webcitation.org/6L69XfzHU

w32. Edrington. Our Company | Edrington [Internet]. 2012 [cited 2013 Dec 1]. Available from: http://edringtongroup.com/our-company#submenu-business-model. Archived at: http://www.webcitation.org/6LXTPN4QF.

w33. The Robertson Trust. About Us [Internet]. 2013 [cited 2013 Dec 31]. Available from: http://www.therobertsontrust.org.uk/index.php/about/. Archived at: http://www.webcitation.org/6MHAZU6ty

w34. British Institute of Innkeeping. Corporate Patrons - BII [Internet]. 2013 [cited 2013 Nov 25]. Available from: http://www.bii.org/membership/Corporate-membership/corporatemembers/current-patrons. Archived at: http://www.webcitation.org/6LPp8YgLE.

w35. British Institute of Innkeeping. Corporate Members - BII [Internet]. 2013 [cited 2013 Nov 25]. Available from: http://www.bii.org/membership/Corporate-membership/corporatemembers/corporate-members. Archived at: http://www.webcitation.org/6LPpFQ1Gs.

w36. Addaction. Addaction the services we offer [Internet]. 2012. Available from: http://www.addaction.org.uk/page.asp?section=111&search=. Archived at: http://www.webcitation.org/6MHB7wIKt.

w37. Third Sector. Business partner: Addaction and Heineken [Internet]. 2011 [cited 2013 Dec 3]. Available from: http://www.thirdsector.co.uk/go/fundraising_good_practice/article/1108794/business-partner-addaction-heineken/. Archived at: http://www.webcitation.org/6LafJ5Dds.

w38. Worldwide Brewing Alliance. Global social responsibility initiatives - “Partnership with Addaction” [Internet]. 2013 [cited 2013 Dec 3]. Available from: http://gsri.worldwidebrewingalliance.org/php/initiatives/initiative.php?ini_id=30. Archived at: http://www.webcitation.org/6Laf4QUtM

w39. The Mentor Foundation International. Annual Report 2012 [Internet]. London; 2012. Archived at: http://www.webcitation.org/6MHBklo8X

w40. Addaction. Government will regret shelving plans to introduce minimum price for alcohol - Addaction [Internet]. 2013 [cited 2013 Dec 31]. Available from: http://www.addaction.org.uk/news.asp?itemid=1162&itemTitle=Government+will+regret+shelving+plans+to+introduce+minimum+price+for+alcohol&section=253&sectionTitle=Press+releases&from=&to=postComment=NO. Archived at: http://www.webcitation.org/6MHCM3Z01.

w41. Mentor UK. Position Statement – Responsibility Deal [Internet]. 2013 [cited 2013 Dec 31]. Available from: http://www.mentoruk.org.uk/2013/08/position-statement-responsibility-deal/. Archived at: http://www.webcitation.org/6MHCVCk0Z.

w42. Diageo Foundation. Diageo Fondation Report and Accounts 2012 [Internet]. London; 2012. Archived at: http://www.webcitation.org/6L4dMPreA

w43. Gannochy Trust. A K Bell and the Gannochy Trust [Internet]. 2013 [cited 2013 Nov 18]. Available from: http://www.gannochytrust.org.uk/index.php?page=a-k-bell. Archived at: http://www.webcitation.org/6LDimpUxB.

w44. Diageo. Diageo Foundation [Internet]. 2013 [cited 2013 Nov 18]. Available from: http://www.diageo.com/en-row/csr/community/Pages/diageo-foundation.aspx. Archived at: http://www.webcitation.org/6LE2psdwn.

w45. Diageo. Diageo Response to the Health and Sports Committee’s Call for Evidence onthe Alcohol (Minimum Pricing) (Scotland) Bill. 2011; Archived at: http://www.webcitation.org/6LXVXBQ6y

w46. House of Commons Health Committee. House of Commons - Written Evidence from Diageo on Governments Alcohol Strategy [Internet]. 2012. Available from: http://www.publications.parliament.uk/pa/cm201213/cmselect/cmhealth/132/132vw31.htm. Archived at: http://www.webcitation.org/6LXWlMJVU.

w47. International Harm Reduction Association. International harm Reduction Association Report and Financial Statements 2010 [Internet]. London; 2010. Archived at: http://www.webcitation.org/6LQ5o05kF

w48. International Harm Reduction Association. International Harm Reduction Association Report and Financial Statement 2008 [Internet]. London; 2008. Archived at: http://www.webcitation.org/6LQ6xH8x3

w49. Harm Reduction International. History | Harm Reduction International [Internet]. 2013 [cited 2013 Nov 25]. Available from: http://www.ihra.net/history. Archived at: http://www.webcitation.org/6LQ84HZnT.

w50. International Center for Alcohol Policies. New Alcohol Harm Reduction Website and Network [Internet]. 2013 [cited 2013 Dec 31]. Available from: http://www.icap.org/AboutICAP/PolicyApproach/TargetedInterventions/IHRA/tabid/202/Default.aspx. Archived at: http://www.webcitation.org/6MHMBvicm.

w51. Alcohol Concern. Annual Report and Financial Statements for the Year Ended 31 March 2012 [Internet]. 2012. Archived at: http://www.webcitation.org/6L3QozY76.

w52. Alcohol Focus Scotland. Annual Report 2012/13 [Internet]. Glasgow; 2013. Archived at: http://www.webcitation.org/6LDhSAUWf.

w53. The Society for the Study of Addiction. The Society for the Study of Addiction Financial Statements 2013 [Internet]. 2013. Archived at: http://www.webcitation.org/6LOmmLLCl.

w54. Alcohol Research UK. Alcohol Research UK Report & Financial Statements For the 16 month period ended 31 March 2012 [Internet]. London; 2012. Archived at: http://www.webcitation.org/6LPwowO2o.

w55. Alcohol Concern. Extract from Alcohol Concern Web Site [Internet]. 2013 [cited 2013 Nov 19]. Available from: http://www.alcoholconcern.org.uk/fundraise/our-funders. Archived at: http://www.webcitation.org/6LFABjuzt.

w56. Alcohol Focus Scotland. Alcohol Focus Website - Background [Internet]. 2013 [cited 2013 Nov 18]. Available from: http://www.alcohol-focus-scotland.org.uk/background. Archived at: http://www.webcitation.org/6LDfqYZVo.

w57. Alcohol Research UK. How we fund our grants [Internet]. 2013 [cited 2013 Nov 25]. Available from: http://alcoholresearchuk.org/grants/how-we-fund-our-grants/. Archived at: http://www.webcitation.org/6LPx45d4s.

w58. Alcohol Concern. Alcohol Concern report and financial statements for the year ended 31 March 2011 [Internet]. 2011. Archived at: http://www.webcitation.org/6L3RHuZOA.

w59. BBC. BBC News - Comic Relief money invested in arms and tobacco shares [Internet]. 2013 [cited 2013 Dec 31]. Available from: http://www.bbc.co.uk/news/uk-25273024. Archived at: http://www.webcitation.org/6MH70ELj5.
